# 84% efficiency improvement in all-inorganic perovskite light-emitting diodes assisted by a phosphorescent material†

**DOI:** 10.1039/c7ra13231j

**Published:** 2018-04-26

**Authors:** Chun-Hong Gao, Xing-Juan Ma, Yue Zhang, Fu-Xing Yu, Zi-Yang Xiong, Zhi-Qiang Wang, Run Wang, Ya-Lan Jia, Dong-Ying Zhou, Zu-Hong Xiong

**Affiliations:** School of Physical Science and Technology, MOE Key Laboratory on Luminescence and Real Time Analysis, Southwest University Chongqing 400715 China gch0122@swu.edu.cn; Soochow Institute for Energy and Materials Innovations (SIEMIS), College of Physics, Optoelectronics and Energy, Soochow University Suzhou 215006 China dyzhou@suda.edu.cn

## Abstract

A novel mixed perovskite emitter layer is applied to design all-inorganic cesium lead halide perovskite light-emitting diodes (PeLEDs) with high electroluminescence (EL) performance, by combining CsPbBr_3_ with iridium(iii)bis[2-(4′,6′-difluorophenyl)pyridinato-N,C^2^′]-picolinate (FIrpic), where FIrpic is a phosphorescent material with very high internal quantum efficiency (IQE) approaching 100%. The CsPbBr_3_:FIrpic PeLEDs show a maximum luminance of 5486 cd m^−2^, and an external quantum efficiency of 0.47%, which are 1.84 and 1.76 times that of neat CsPbBr_3_ PeLEDs, respectively. It is found that FIrpic molecules as an assistant dopant can efficiently transmit energy from the excitons of FIrpic to the excited state of the CsPbBr_3_ emitter *via* a Förster energy transfer process, leading to enhanced EL efficiency in the CsPbBr_3_:FIrpic PeLEDs.

## Abstract 2

Remarkable EL performance is achieved in CsPbBr_3_:FIrpic perovskite light-emitting diodes assisted by a phosphorescent material.

## Graphical abstract

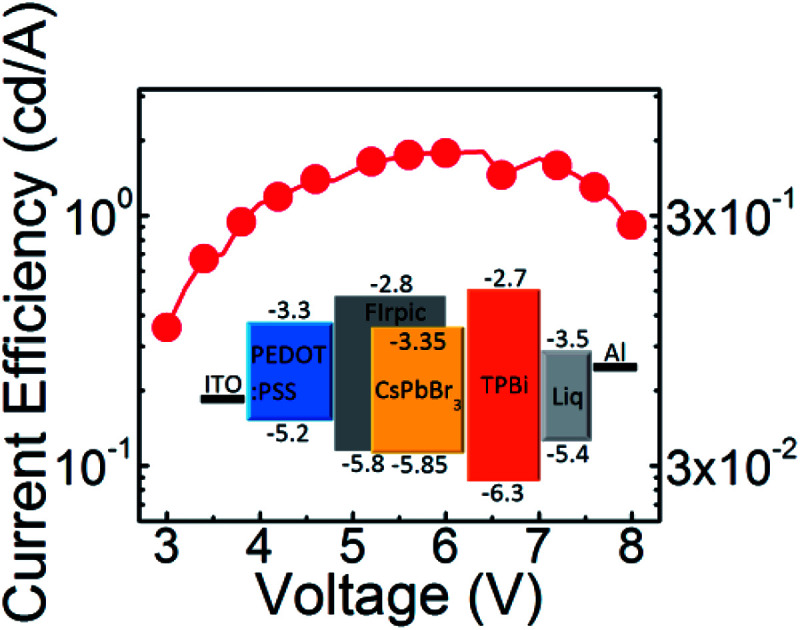

## 1. Introduction

In recent years, all-inorganic halide perovskite light-emitting diodes (PeLEDs) have attracted a lot of attention due to their high photoluminescence (PL) quantum yield, high color purity, better thermal stability and higher charge-carrier mobility than organic–inorganic perovskites.^1–4^ The first perovskite light-emitting diode observed at room-temperature was reported by R. H. Friend's group in 2014,^5^ showing the highest luminescence of 364 cd m^−2^, external quantum efficiency (EQE) of 0.1% for green PeLEDs, and a tunable luminescence spectrum. And Yantara *et al.* presented the first CsPbBr_3_ LED, with enhanced maximum luminescence (407 cd m^−2^), but the current efficiency (CE, 0.035 cd A^−1^) and external quantum efficiency (EQE, 0.008%) were still low.^6^ To improve the performance, much work has focused on obtaining complete surface coverage, efficient charge injection, and better carrier transporting balance, such as interfacial engineering,^7–11^ crosslinking method,^2^ polymer-assisted method,^12,13^ small organic molecule-assisted method.^14–16^ And many researchers have achieved excellent PeLEDs performance by doping emitter materials (such as poly(ethylene glycol) (PEO)),^12,13,17^ hole transporting materials (such as 1,3-bis(9-carbazolyl) benzene (mCP)),^14^ electron transporting materials (such as 1,3,5-tris(1-phenyl-1*H*-benzimidazol-2-yl)benzene (TPBi)),^15^ 1,3,5-tri(*m*-pyridin-3-yl-phenyl)benzene (TmPyPB),^16^ and bipolar transporting materials (such as PVK:TPBi),^18^ which can be attributed to the improvement of the film coverage, the charge injection and the efficient energy transfer of singlet excitons from the assistant dopants to the excited state of the perovskite emitter. However, the triplet excitons in these PeLEDs are wasted, since all the triplet excitons would decay non-radiatively due to the spin-forbidden of triplet exciton transition in fluorescent emitters.^19^ Usually, to break the limitation in internal quantum efficiency of fluorescent LEDs (<25%), heavy transition metal atoms are brought in the phosphorescent molecules to harvest both singlets and triplets due to the strong spin orbit coupling in organic light-emitting diodes (OLEDs).^19–21^ Thus, organotransition metal compounds have been extensively studied in efficient organic emitting diodes (OLEDs) to achieve 100% internal quantum efficiency. Iridium(iii)bis[2-(4′,6′-difluorophenyl)pyridinato-N,C^2^′] picolinate (FIrpic), a phosphorescent material with very high internal quantum efficiency (IQE) approaching to 100%, is wildly used in OLEDs as a blue emitter and shows good EL performance.

In this work, FIrpic is adopted as an assistant to form CsPbBr_3_:FIrpic composite film. The PeLEDs based on this composite emissive layer can harvest both singlet and triplet excitons. And a maximum luminance of 5486 cd m^−2^, a maximum current efficiency of 1.80 cd A^−1^, and a maximum EQE of 0.47% are obtained.

## 2. Experimental section

### 2.1. Materials and perovskite precursor solution preparation

CsPbBr_3_ precursor solutions were made by dissolving PbBr_2_ (Alfa Aesar, 99.999%) and CsBr (Alfa Aesar, 99.999%) in dimethylsulfoxide (DMSO) with equimolar molar ratio at 10 wt% iridium(iii)bis[2-(4′,6′-difluorophenyl)pyridinato-N,C^2^′] picolinate (FIrpic, >99.5%) was dissolved in DMSO at 1 mg ml^−1^. The two solutions were stirred for 12 hours at room temperature in a glove box, separately. Before spin-coating, the solutions were mixed with different proportions and stirred continually for 4 hours. The materials including poly(3,4-ethylenedioxythiophene):poly(*p*-styrene sulfonate) (PEDOT:PSS, Heraeus, Clevios AI 4083) are bought from Shanghai Han Feng Chemical Co. Ltd. 2,2′,2′′-(1,3,5-benzinetriyl)-tris(1-phenyl-1*H*-benzimidazole) (TPBi, >99%), 8-hydroxyquinolinato lithium (Liq, >99%), and aluminum (Al, >99%) were purchased from Suzhou Fangsheng Photoelectricity Shares Co, Ltd. All the materials used in this study were obtained commercially and used as received without further purification.

### 2.2. PeLEDs fabrication

Patterned indium-tin oxide (ITO) glass substrates were cleaned successively using deionized water, ethanol, and acetone, and dried in an oven at 100 °C for 10 min. After 5 min treatment with ultraviolet (UV)–ozone plasma, PEDOT:PSS was spin-coated onto ITO glass substrates at 4500 rpm for 40 s and annealed in air at 120 °C for 20 min. Then, the samples were placed in a nitrogen-filled glovebox for 30 min to cool down. The perovskite film was prepared by one-step spin-coating the perovskite precursor solution at 4000 rpm for 60 s and placed in a low vacuum environment of −1 bar for 20 min to remove residual DMSO solvent. Finally, TPBi (65 nm), Liq (2.5 nm), and Al (120 nm) were sequentially deposited by vacuum thermal depositing system, under a high vacuum (≤5 × 10^−4^ Pa). Preparation of the perovskite films, and encapsulation of PeLEDs were carried out in a nitrogen-filled glove box. The active area of the device was 6 mm^2^.

### 2.3. Measurements and characterizations

We used the UV-vis spectrophotometer (Shimadzu UV-2600) and Fluorolog-3 luminescence spectroscopy to measure the absorption and PL spectra, respectively. The XRD pattern and time-resolved PL spectra were acquired using a TD-3500 X-ray diffractometer and fluorescence spectrometer (Fluorolog-3) severally. The PR670 spectrophotometer was used to collect the EL spectra. The current density–luminance–voltage (*J*–*L*–*V*) characterizations and stability of all PeLEDs were carried out by a light-emitting diode measurement system, including a Keithley2400 source meter, and a calibrated Si photodiode (Photoelectric Instrument Factory of Beijing Normal University, ST-86LA). All measurements were carried out at room temperature under ambient conditions.

## 3. Results and discussion

### 3.1. PeLEDs performance

**Fig. 1:**
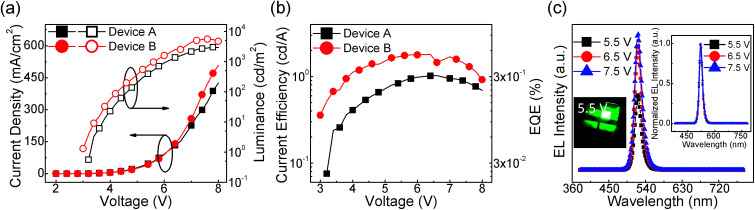
(a) Current density–voltage–luminance (*J*–*V*–*L*), (b) current efficiency–voltage–external quantum efficiency (CE–*V*–EQE) of Devices A (neat CsPbBr_3_) and B (CsPbBr_3_:FIrpic 0.5 mg ml^−1^). (c) EL intensity–wavelength curves of Device B under different driving voltages of 5.5, 6.5, and 7.5 V. The insets show an emission photo of Device B at driving voltage of 5.5 V (left inset) and the normalized EL spectra of this PeLED under different driving voltages of 5.5, 6.5, and 7.5 V (right inset).

Using the CsPbBr_3_:FIrpic films as the emissive layers, bright and efficient PeLEDs are demonstrated with a typical layered architecture of ITO (120 nm)/PEDOT:PSS (30 nm)/CsPbBr_3_:FIrpic (*X* mg ml^−1^)/TPBi (65 nm)/Liq (2.5 nm)/Al (120 nm), where “*X*” are equal to 0, 0.5 for Devices A and B. The concentration optimization of FIrpic are shown in ESI Fig. S1.† The current density–voltage–luminance (*J*–*V*–*L*) and current efficiency–voltage–external quantum efficiency (CE–*V*–EQE) characteristics are shown in Fig. 1(a) and (b). Device B with FIrpic exhibits better EL performance than Device A with higher current density, luminance, current efficiency and EQE at each applied voltage, and shows maximum luminance of 5486 cd m^−2^, maximum current efficiency of 1.80 cd A^−1^, and maximum EQE of 0.47%, which are 1.84, 1.76 and 1.76 times to that of the Device A (2974 cd m^−2^, 1.02 cd A^−1^, and 0.26%), respectively. The corresponding characteristics data about the repeatability of the devices are summarized in Table S1.† The Device B emits green light from only CsPbBr_3_ despite of FIrpic (peaked at 470 nm)^22^ with high color purity of FWHM ∼18 nm and a peak intensity located at 522 nm, as shown in Fig. 1(c). In the right inset of Fig. 1(c), it could be found that the shapes of EL spectra are rarely affected by the different applied voltages. In addition, the EL spectra of each devices with different FIrpic concentrations are shown in ESI Fig. S2† and similar results are exhibited. These results reveal that the energy transfer from FIrpic to CsPbBr_3_ was efficient and complete. And the detailed mechanism to improve the EL performance in Device B are investigated by the following sections.

### 3.2. Characterization of perovskite film

**Fig. 2:**
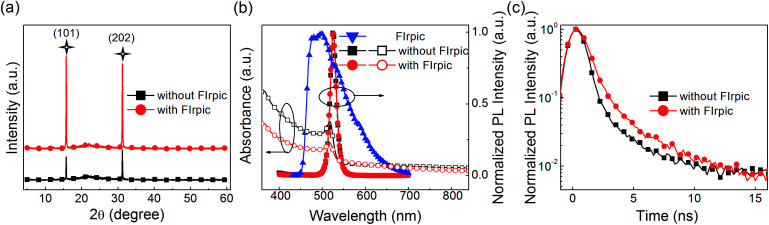
(a) XRD patterns, (b) UV-vis absorption and normalized PL spectra, (c) time-resolved PL spectra of CsPbBr_3_ film without and with FIrpic (0.5 mg ml^−1^).

In order to investigate the physical mechanism of the enhanced EL performance in the PeLEDs, the XRD pattern, absorption and PL spectra, and the time-resolved PL spectra of the CsPbBr_3_ films FIrpic (0.5 mg ml^−1^) are exhibited in Fig. 2(a). In Fig. 2(a), by comparing the intensity changes of peaks at 15.7° and 31.1° assigned to the (101) and (202) planes of perovskite structure in the XRD pattern of both films, it can conclude that peaks of CsPbBr_3_ film with FIrpic are sharper and more intense, which demonstrate that the presence of FIrpic can induce the crystal growth along the (101) and (202) planes and may do good to the EL performance.^8,10,13^ And the XRD patterns match well with orthorhombic crystal structure.^13^ The top-view and cross-sectional SEM images of the neat CsPbBr_3_ film and the CsPbBr_3_:FIrpic film (0.5 mg ml^−1^) are shown in Fig. S3 and S4,† respectively. It can be found that coverage is improved in the CsPbBr_3_:FIrpic film. And the thickness of both the neat CsPbBr_3_ film and CsPbBr_3_:FIrpic film (0.5 mg ml^−1^) are estimated to ∼30 nm. UV-vis absorption and PL emission spectra of CsPbBr_3_ film with 0 and 0.5 mg ml^−1^ FIrpic are plotted in Fig. 2(b). The two kinds of films exhibit similar shapes of absorption spectra (centered at ∼518 nm) and PL spectra (peaked at ∼525 nm with a narrow FWHM of ∼18 nm). It is worthwhile mentioning that sufficient spectral overlap between the absorption spectra of the CsPbBr_3_ emitter and the PL spectra of the assistant dopant FIrpic, which is beneficial to energy transfer from FIrpic to CsPbBr_3_. And the shape of PL spectra of CsPbBr_3_:FIrpic film is almost same to the neat CsPbBr_3_ film without the sub-peak from FIrpic, which may demonstrate that the energy transfer from FIrpic to the CsPbBr_3_ are effective and complete. The FIrpic-doped CsPbBr_3_ film presents a much longer PL lifetime than that of the neat CsPbBr_3_ film, which is revealed by the time-resoled PL spectra (Fig. 2(c)). The average lifetime (*τ*_avg_) extracted from the PL decay curve for the composite perovskite film is about 1.69 ns, while the neat CsPbBr_3_ has a shorter lifetime of 0.82 ns, which imply that the FIrpic additive can effectively reduce non-radiation recombination and result in enhanced the EL performance of PeLEDs. The PL curves can be fitted by the following eqn (1):1
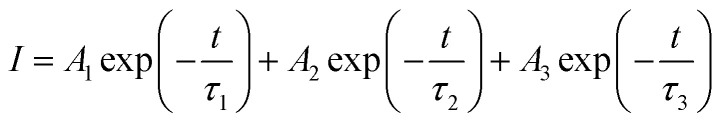


The average lifetime (*τ*_avg_) of the entire decay process can be calculated by the following formula (2):2
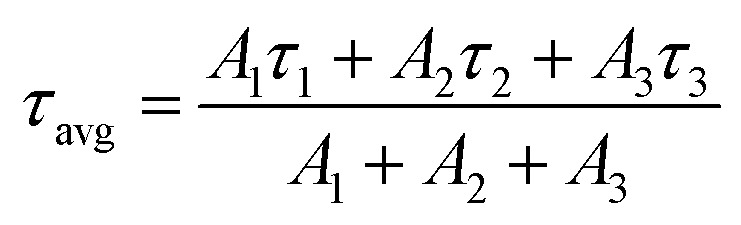


*τ*_1_, *τ*_2_ and *τ*_3_ are the lifetimes of the three decay components; and *A*_1_, *A*_2_, and *A*_3_ are the fractions of the three decay components, respectively. According to the study by Zheng *et al.*,^23^ the *τ*_3_ (slow) decay component is related to radiative recombination inside the grains, while the *τ*_1_ (fast) and *τ*_2_ (middle) decay components are attributed to “two kinds of trap-assisted recombination at grain boundaries.” The fitting parameters are collected in Table 1. It can be found that the proportions of *τ*_1_ and *τ*_2_ are reduced, which suggests that traps in the FIrpic assisted CsPbBr_3_ film are reduced, due to the effective passivation brought in by the FIrpic assistant. Moreover, the lifetime of *τ*_3_ is extended and the its proportion is improved, which suggests that the radiative recombination inside the grains are enhanced, thus leading to the better EL performance in the CsPbBr_3_:FIrpic based PeLEDs compared to the one without the FIrpic assistant.^17^

**Table 1:** Multi-exponential fitting parameters for PL decays both neat CsPbBr_3_ and CsPbBr_3_:FIrpic (0.5 mg ml^−1^) films

Films	*A*_1_ (%)	*τ*_1_ (ns)	*A*_2_ (%)	*τ*_2_ (ns)	*A*_3_ (%)	*τ*_3_ (ns)	*τ*_avg_ (ns)
CsPbBr_3_:FIrpic (0 mg ml^−1^)	85.08	0.38	10.65	1.65	4.27	7.56	0.82
CsPbBr_3_:FIrpic (0.5 mg ml^−1^)	78.22	0.64	16.17	2.76	5.61	13.23	1.69

### 3.3. Energy transfer process

**Fig. 3:**
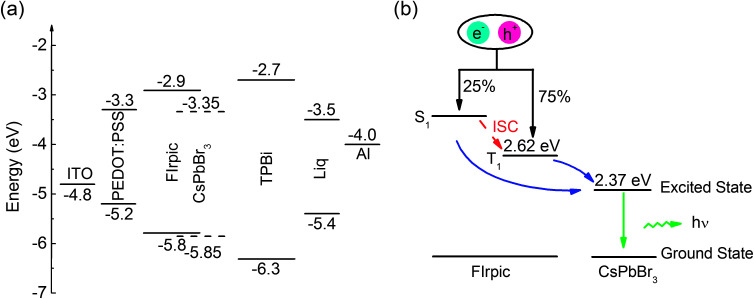
(a) Energy level diagram of Device B, (b) energy transfer scheme for the CsPbBr_3_:FIrpic emitter layer (Förster energy transfer process is indicated by blue solid arrows).

The energy level diagram of each layer of Device B is shown in Fig. 3(a), and all energy level values are taken from the literature.^14,24–26^ There are two exciton generating interfaces, which are located at the PEDOT:PSS/CsPbBr_3_:FIrpic interface and CsPbBr_3_:FIrpic/TPBi interface, due to no injection barrier for electrons injected from TPBi to CsPbBr_3_:FIrpic film and high injection barrier for holes at PEDOT:PSS/CsPbBr_3_:FIrpic interface (0.6 eV) and CsPbBr_3_:FIrpic/TPBi interface (0.45 eV). And excitons can also be generated on FIrpic and CsPbBr_3_. The energy transfer diagram is described schematically in Fig. 3(b). As shown in Fig. 3(b), excitons generated in FIrpic with 25% singlets (S_1_) and 75% triplets (T_1_). And in FIrpic, the energy of S_1_ of FIrpic can be transfer to T_1_ through inter system crossing process (ISC). And the energy of both S_1_ and T_1_ of FIrpic can be transferred to the excited state of CsPbBr_3_*via* Förster energy transfer process^21^ and thus leading to potentially 100% IQE in CsPbBr_3_:FIrpic PeLEDs.

### 3.4. The stability of PeLEDs

**Fig. 4:**
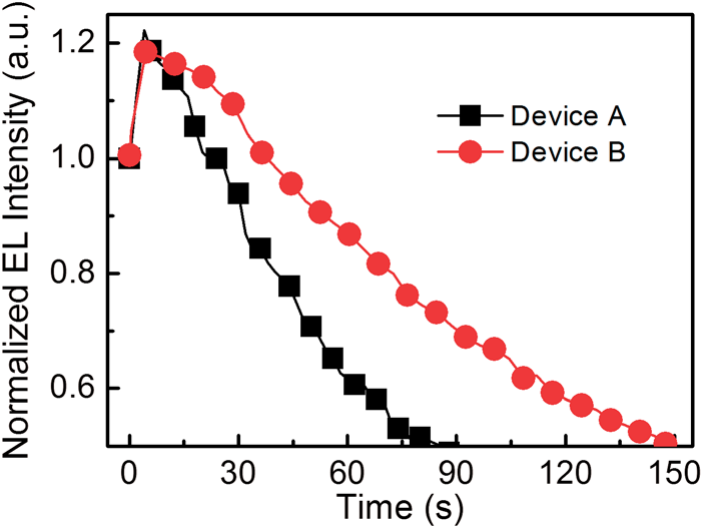
The stability of Device A (with a neat CsPbBr_3_ film) and Device B (with FIrpic-doped CsPbBr_3_ film).

In order to further reveal the effect of FIrpic, the half lifetimes of PeLEDs (Devices A and B) are performed and are shown in Fig. 4. The half lifetime is the time duration from the initial luminance of 100 cd m^−2^ decreasing to the half luminance of the initial luminance. Device B (with CsPbBr_3_:FIrpic film) exhibit a much longer half lifetime of 147 s, which is 1.6 times than that of Device A (87 s). The better stability of Device B may be benefit from reduced current leakage owing to the better coverage^9,12,13,15^ and enhanced radiative recombination inside the grain due to the reduced traps, better passivation and higher exciton harvesting efficiency in the in CsPbBr_3_:FIrpic film compared to that of Device A.

## 4. Conclusion

In summary, the enhancements of EL performance and stability can be attributed three factors in the CsPbBr_3_:FIrpic PeLEDs. Firstly, the orientation of the crystallization could be remarkably enhanced by FIrpic along with the reduced traps, better passivation in the CsPbBr_3_:FIrpic film. Second, FIrpic improves the internal quantum efficiency in CsPbBr_3_:FIrpic, due to almost 100% IQE of FIrpic which can permit efficient transfer of all electrically excitons from the assistant dopant (FIrpic) to the perovskite emitter (CsPbBr_3_) *via* Förster energy transfer process. And last, the higher coverage of the CsPbBr_3_:FIrpic benefit to reduce current leakage. The CsPbBr_3_:FIrpic composite film provide a facile method to achieve highly efficient PeLEDs and a new route for advanced light emission applications.

## Conflicts of interest

The authors declare no conflicts of interest.

## Supplementary Material

RA-008-C7RA13231J-s001
