# Supplementary material for: 84% efficiency improvement in all-inorganic perovskite light-emitting diodes assisted by a phosphorescent material
Source: RSC Adv. 2018 Apr 26;8(28):15698–702. doi: 10.1039/c7ra13231j (PMC9080124; doi:10.1039/c7ra13231j)
Supplement: RA-008-C7RA13231J-s001 [file RA-008-C7RA13231J-s001.pdf]

Electronic Supplementary Material (ESI) for RSC Advances.
This journal is © The Royal Society of Chemistry 2018

Supporting Information

84% efficiency improvement in all-inorganic perovskite light-emitting diodes assisted by a phosphorescent material

Chun-Hong Gao[a†]*, Xing-Juan Ma[a†], Yue Zhang[a], Fu-Xing Yu[a], Zi-Yang Xiong[a], Zhi-Qiang Wang[a], Run Wang[a], Ya-Lan Jia[a], Dong-Ying Zhou[b], Zu-Hong Xiong[a]*

[a]School of Physical Science and Technology, MOE Key Laboratory on Luminescence and Real Time Analysis, Southwest University, Chongqing, 400715, China. E-mail: gch0122@swu.edu.cn

[b]Soochow Institute for Energy and Materials Innovations (SIEMIS), College of Physics, Optoelectronics and Energy, Soochow University, Suzhou 215006, China. E-mail: dyzhou@suda.edu.cn

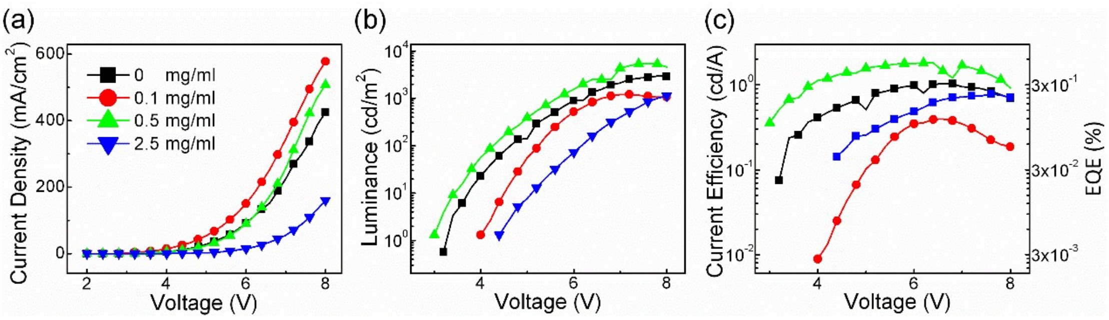

Figure S1. EL performances of devices with different concentrations of FIrpic: (a) current density-voltage (*J*-*V*); (b) luminance-voltage (*L*-*V*); (c) current efficiency-voltage-EQE (CE-*V*-EQE) of four PeLEDs with structured as: ITO (120 nm)/PEDOT:PSS (30 nm)/$CsPbBr_3$:FIrpic (X mg/ml)/TPBi (65 nm)/Liq (2.5 nm)/ Al (120 nm), where "X" stands for 0, 0.1, 0.5, 2.5.

Table S1. Summary of performances for 5 PeLEDs in a single batch with composite emitter layer $CsPbBr_3$:FIrpic (0.5 mg/ml).

Device No.	$V_{(turn-on)}$ (V)	L_{max} (cd/m^2)[b]	CE_{max} (cd/A)[c]	EQE_{max} (%)[d]
1	3.0	5486	1.80	0.47
2	2.8	5214	1.64	0.43
3	2.8	6470	1.68	0.44
4	3.3	4510	1.87	0.49
5	3.0	3093	1.54	0.40

[a] Turn-on voltage at 1 cd/m^2.

[b] Maximum luminance.

[c] Maximum current efficiency.

[d] Maximum external quantum efficiency.

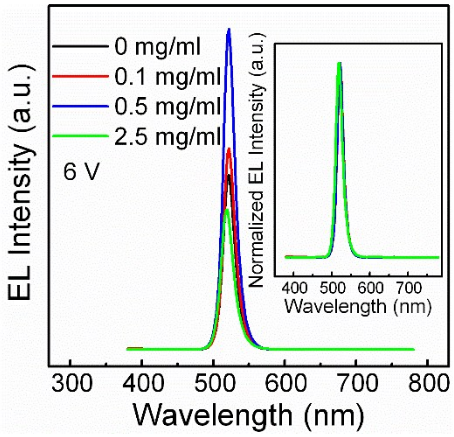

Figure S2. EL spectra and the Normalized EL spectra of the PeLEDs with different concentrations of FIrpic under the driving voltages of 6 V. The four PeLEDs are structured as: ITO (120 nm)/PEDOT:PSS (30 nm)/$CsPbBr_3$:FIrpic (X mg/ml)/TPBi (65 nm)/Liq (2.5 nm)/ Al (120 nm), where "X" stands for 0, 0.1, 0.5, 2.5.

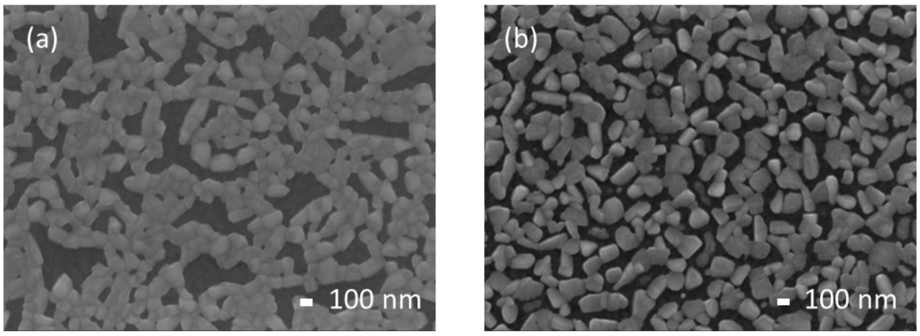

Figure S3. Top-view SEM images of perovskite films of (a) pristine $CsPbBr_3$, (b) $CsPbBr_3$ with FIrpic (0.5 mg/ml). It can be found, higher coverage is achieved in the perovskite film with suitable concentration of FIrpic (0.5 mg/ml) added in the $CsPbBr_3$ (77.7%, estimated from SEM images by using ImageJ software) than the one without FIrpic (76.1%), which benefit to the EL efficiency of PeLEDs.

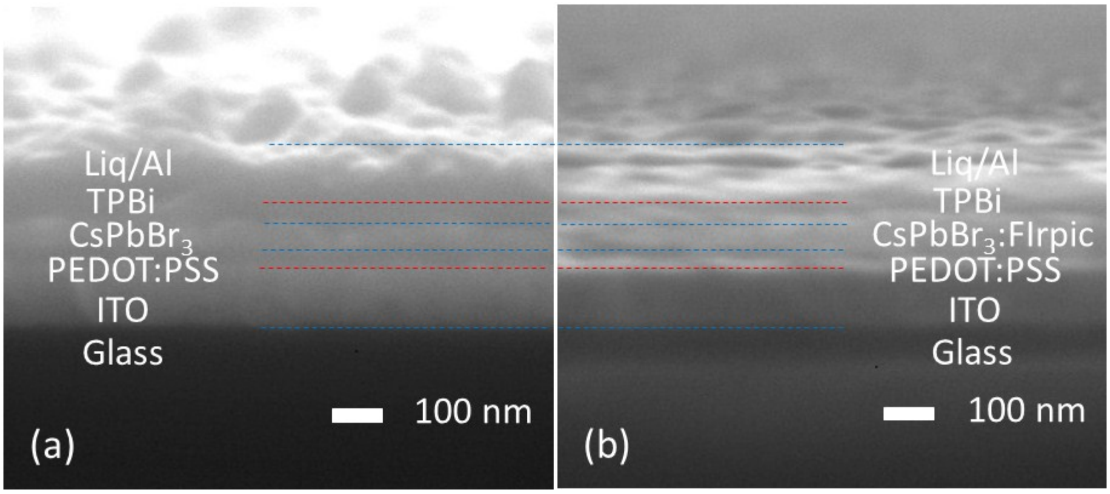

Figure S4. The cross-sectional SEM images of neat $CsPbBr_3$ and $CsPbBr_3$:FIrpic (0.5 mg/ml) based PeLED. The thickness of both the neat $CsPbBr_3$ film and $CsPbBr_3$:FIrpic film (0.5 mg/ml) are estimated to ~30 nm.
